# Efficacy and safety analysis of midazolam combined with dezocine sedation and analgesia colonoscopy in patients with inflammatory bowel disease: a prospective single-center open study

**DOI:** 10.3389/fphar.2023.1150045

**Published:** 2023-07-10

**Authors:** Yongpeng Chen, Yi Lu, Xueyuan Xiang, Liping Fu, Yanan Liu, Chujun Li, Jiachen Sun

**Affiliations:** ^1^ Department of Gastrointestinal Endoscopy, The Sixth Affiliated Hospital, Sun Yat-sen University, Guangzhou, China; ^2^ Guangdong Provincial Key Laboratory of Colorectal and Pelvic Floor Diseases, The Sixth Affiliated Hospital, Sun Yat-sen University, Guangzhou, China; ^3^ Biomedical Innovation Center, The Sixth Affiliated Hospital, Sun Yat-sen University, Guangzhou, China

**Keywords:** inflammatory bowel disease, midazolam, dezocine, perforation, colonoscopy

## Abstract

**Objective:** Colonoscopy plays an important role in the diagnosis, prognosis prediction, assessment of disease activity and severity, and treatment of inflammatory bowel disease (IBD)-related complications. However, some patients refuse to undergo colonoscopy due to perceived pain and other discomfort, their diagnosis and treatment are affected. Therefore, we conducted a prospective study to explore the efficacy and safety of midazolam combined with dezocine for sedation in IBD patients undergoing colonoscopy.

**Methods:** 224 patients were divided into sedative-colonoscopy-group (SCG, n = 93), anesthesia-colonoscopy-group (ACG, n = 90) and ordinary-colonoscopy-group (OCG, n = 41). The vital signs (blood pressure, pulse, respiration and blood oxygen saturation), pain degree during colonoscopy, satisfaction and complication rates of the three groups were compared.

**Results:** Before colonoscopy, there was no significant difference among the vital signs of the three groups. The vital signs of the ACG were significantly lower than those of the SEG and the OCG (*p* < 0.05), and the difference was not significant between the SCG and OCG during colonoscopy. The colonoscopy pain score in the SCG was lower than that in the OCG (0.79 ± 1.09 vs. 2.98 ± 1.27, *p* < 0.001). The satisfaction score of the SCG (9.26 ± 1.16) was not significantly different from that of the ACG (9.42 ± 1.41) but was higher than that of the OCG (6.63 ± 1.13) (*p* < 0.001). The total complication rate of the ACG was 45.56% (41/90), which was significantly higher than that of the SCG [20.43% (19/93)] and the OCG [19.51% (8/41)]. Colon perforation, abnormal blood pressure fluctuation and hypoxemia were significantly more common in the ACG than in the SCG and the OCG (*p* < 0.05). However, there was no significant difference in the incidence of complications between the SCG and OCG.

**Conclusion:** Compared with ordinary-colonoscopy, colonoscopy performed under midazolam and dezocine sedation is more comfortable for patients, thereby increasing satisfaction and compliance. Colonoscopy that is performed under midazolam and dezocine is similar to colonoscopy that is anesthesia with propofol in terms of comfort, satisfaction and compliance and similar to ordinary-colonoscopy in terms of safety. Considering the shortage of anesthesiologists, the application of midazolam combined with dezocine for digestive endoscopy is worthy of clinical promotion.

## Introduction

Inflammatory bowel disease (IBD) mainly refers to two major types of chronic recurrent inflammatory bowel disease: Crohn’s disease (CD) and ulcerative colitis (UC) (Gajendran et al., 2018). Epidemiological population studies have shown that the prevalence of IBD in Western countries is approximately 0.5% (Goldstone and Steinhagen, 2019). The annual incidence rate of CD in North America is 3.1–20.2/100000 people. The incidence rate of CD in Japan, Hong Kong and South Korea is also increasing. The incidence rate of CD in Eastern countries is reported to be 54/100000 (Gajendran et al., 2018). IBD is chronic condition that is associated with many complications and the need for surgical intervention because of its negative impact on quality of life, considerable pain, heavy financial burden to the patients and risk of death (Gajendran et al., 2018; Goldstone and Steinhagen, 2019). Therefore, early diagnosis, standardized treatment and long-term monitoring of intestinal conditions are very important for the prognosis of IBD patients (Navaneethan et al., 2011). Colonoscopy plays an important role in the diagnosis, prognosis prediction (such as mucosal healing), evaluation of disease activity and severity, identification of superimposed infections (such as cytomegalovirus infection and *Clostridium difficile* infection), and treatment of IBD-related complications (Navaneethan et al., 2011). However, some patients refuse the examination or interrupt the examination and treatment due to pain and other discomfort, thereby affecting the quality of the examination, accuracy of the diagnosis and selection of treatment (Xiao et al., 2016).

Intravenous infusion of propofol for anesthesia can improve the patients’ comfort level and compliance during colonoscopy (Adams et al., 2017). However, the treatment window following anesthesia induction with propofol is narrow, which may lead to fluctuations in sedation depth and the occurrence of cardiopulmonary complications. Because professional anesthesiologists who are trained in airway management must continuously monitor the patient, the labour cost is increased and the demand for colonoscopy cannot be met, thus delaying the diagnosis and treatment of patients (das Neves et al., 2016; Jin et al., 2017). As anesthesia during colonoscopy is a risk factor for perforation during colonoscopy, the mortality rate is increased by 5% (de’Angelis et al., 2018).

Because research shows that sedation during colonoscopy can effectively reduce anxiety, relieve discomfort, and improve patient tolerance and satisfaction, it has been gradually implemented in clinical practice in recent years (das Neves et al., 2016). Sedative and analgesic drugs can be administered by nurses under the guidance of the doctors performing endoscopy, and a combination of benzodiazepines and opioids are used in many countries during colonoscopy (Dossa et al., 2021). However, there is no consensus on drug selection, dosage and time interval of administration. Midazolam is a benzodiazepine that has a fast onset, a short elimination half-life, a small local stimulation effect, a high safety limit, a high treatment index, and an anti-anxiety effect that can induce anterograde amnesia, stabilize haemodynamics, is not accumulated or has any residual effects and has been widely used for sedation despite its unideal analgesic effect (Lee et al., 2021). In many international guidelines, such as those proposed by the German Society for Gastroenterology, Digestive and Metabolic Diseases (GSGMD) and the Spanish Society of Gastrointestinal Endoscopy (SSGE), midazolam is recommended as a first-line drug for inducing sedation in patients undergoing gastrointestinal endoscopy (Igea et al., 2014; Riphaus et al., 2016). Dezocine is an opioid analgesic that has essentially no potential of being addictive and is often used for digestive endoscopy (Xu et al., 2016). A study showed that compared with fentanyl and propofol, the combination of dezocine and propofol for colonoscopy can improve the safety of surgery and reduce the occurrence of adverse reactions (Xu et al., 2016).

Patients with UC are at high risk for colon cancer, so they need to be monitored more closely by colonoscopy. However, frequent colonoscopy exposes these patients to a higher risk of complications, including iatrogenic perforation (DiCaprio et al., 2018). In addition, a study reported that CD is a risk factor related to intestinal perforation caused by colonoscopy (de’Angelis et al., 2018). Although the current data show that the overall risk of perforation is still low, the significant incidence rate associated with this complication, as well as the serious consequences of perforation (Makkar and Shen, 2013), requires us to provide a safe and comfortable colonoscopy. However, there has been no discussion on a safe and comfortable sedation or analgesia plan for IBD patients undergoing colonoscopy. Therefore, we conducted a prospective study to explore the effectiveness and safety of midazolam combined with dezocine for sedation and analgesia in IBD patients undergoing colonoscopy.

## Materials and methods

### Subjects

**FIGURE 1:**
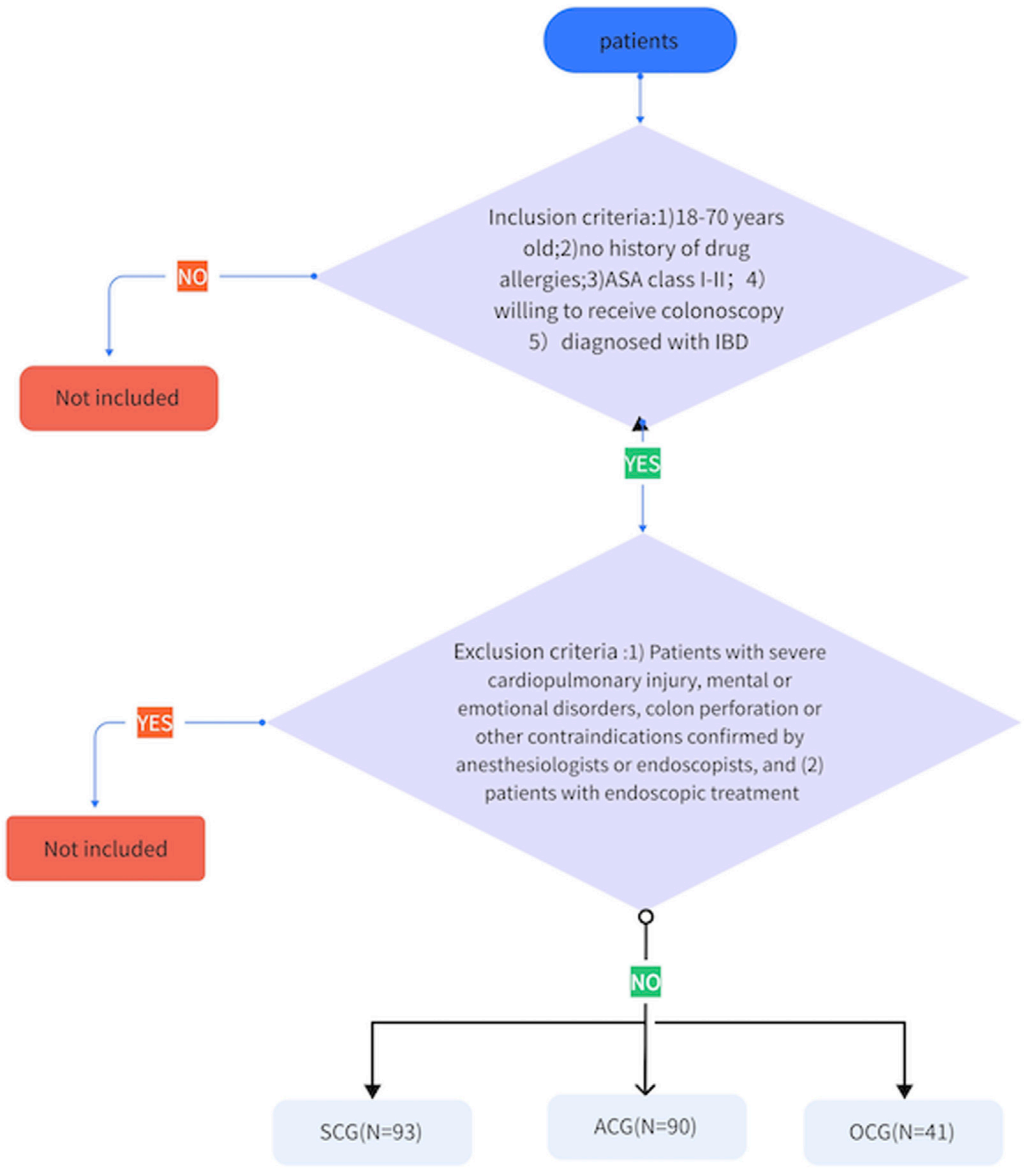
Grouping Process.

Patients who were treated at the Endoscopy Center of the Sixth Affiliated Hospital of Sun Yat-sen University from May 2021 to December 2022 were selected. The inclusion criteria were as follows: 1) IBD patients aged 18–70 years, 2) patients with no drug allergy history, 3) patients with an American Society of Anesthesiologists (ASA) grade I-II, and 4) patients with a diagnosis of IBD. The exclusion criteria were as follows: 1) patients with a severe cardiopulmonary injury, mental or emotional disorders, colon perforation or other contraindications that have been confirmed by anesthesiologists or endoscopic doctors and 2) patients who needed digestive endoscopy. Patients were divided into a sedative colonoscopy group (SCG, n = 93), an anesthesia colonoscopy group (ACG, n = 90) or an ordinary colonoscopy group (OCG, n = 41) (as shown in Figure 1). All colonoscopies were performed by doctors with extensive experience in performing colonoscopy (>2000 cases). This study was approved by the hospital ethics committee (ethics number: 2021ZSLYEC-182), and written informed consent was obtained from all the patients.

### Preoperative preparation

All the patients received the same educational information and were subjected to the same bowel preparation program. Before surgery, they fasted for at least 6 h and did not drink water for at least 2 h.

### Methods of sedation and analysis, and methods of anesthesia

SCG patients were given midazolam (NMPNH10980025, 0.02–0.05 mg/kg) and dezocine (NMPNH20080329, 0.05 mg/kg) intravenously, and additional doses of midazolam and dezocine were given according to pain and vital signs during the operation. ACG patients were only injected with propofol (1.0–2.5 mg/kg) intravenously to induce anesthesia. In clinical practice, some patients undergo anesthesia colonoscopy have received fentanyl combined with propofol, while others only receive propofol. Our experiment was set to only administer propofol in anesthesia colonoscopy. When the eyelash reflex disappeared, the colonoscope was inserted, and the depth of anesthesia was maintained during the operation. Throughout the examination, all patients were provided with supplemental oxygen through an oxygen tube (3 L/min), and the mean arterial pressure (MAP), pulse rate (PR), respiratory rate (RR) and blood oxygen saturation (SpO2) were recorded with an automatic monitor. Compared to propofol, the safe dose range of midazolam and dezocine is relatively large, so sedation and analgesia are administered by endoscopic nurses, who also help monitor the vital signs on electrocardiogram monitoring, following the endoscopic doctor’s instructions (Jin et al., 2017).

### Data record: MAP, PR, RR, and SpO2 were continuously monitored and recorded at 4 time points during the examination

T1, before colonoscopy; T2, when reaching the ileocecal valve; T3, 5 min after colonoscopy; and T4, after colonoscopy. Adverse blood pressure fluctuation is defined as an increase or decrease in MAP greater than 20%. Hypoxemia was defined as peripheral blood oxygen saturation lower than 85% (Wu et al., 2014). A postoperative questionnaire was used to evaluate patients’ satisfaction with digestive colonoscopy and sedation level, overall pain degree, the level of the most intense pain during colonoscopy, dizziness score, and whether the patient would choose the procedure again or recommend the examination method. The subjective score was based on the 11-point numerical rating scale (NRS), with 0 indicating “very dissatisfied” and 10 indicating “very satisfied”. Complications included severe blood pressure fluctuation, hypoxemia, intestinal mucosal injury, perforation and hemorrhage.

### SPSS 21.0 software was used for statistical analysis

All data are expressed as percentages, averages and standard deviations or medians and quartile ranges. The measured data are expressed as (χ±s). The comparison of baseline and clinical characteristics between groups was analyzed by one-way ANOVA or Kruskal‒Wallis one-way ANOVA, and paired comparisons within groups were performed by paired tests. The counting data are expressed as ratios (%), and the chi-square test was used to analyze the differences between groups. All statistical tests were bilateral tests, and P < 0.05 was considered significant.

## Result

### General data

A total of 242 patients (118 males (52.69%) were included in this study, and the average age of the patients was 25.70 ± 4.45 years (as shown in Table 1).

**TABLE 1:** Basic clinical characteristics of the enrolled patients (‾χ±s).

	SCG (N = 93)	ACG (N = 90)	OCG (N = 41)
Sex			
Male	46	47	25
Female	47	43	16
Age	25.61 ± 4.01	25.48 ± 4.02	26.37 ± 6.09
BMI	16.94 ± 2.52	16.92 ± 2.29	16.74 ± 2.29
Education level			
Junior high school and below	9	15	10
Senior high school	23	23	10
University and above	61	52	21
Smoking history			
Yes	7	4	3
No	86	86	38
Drinking history			
Yes	11	4	3
No	82	86	38
History of gastrointestinal surgery			
Yes	16	11	3
No	77	79	38
History of colonoscopy endoscopy			
Yes	90	89	40
No	3	1	1

### Safety

**FIGURE 2:**
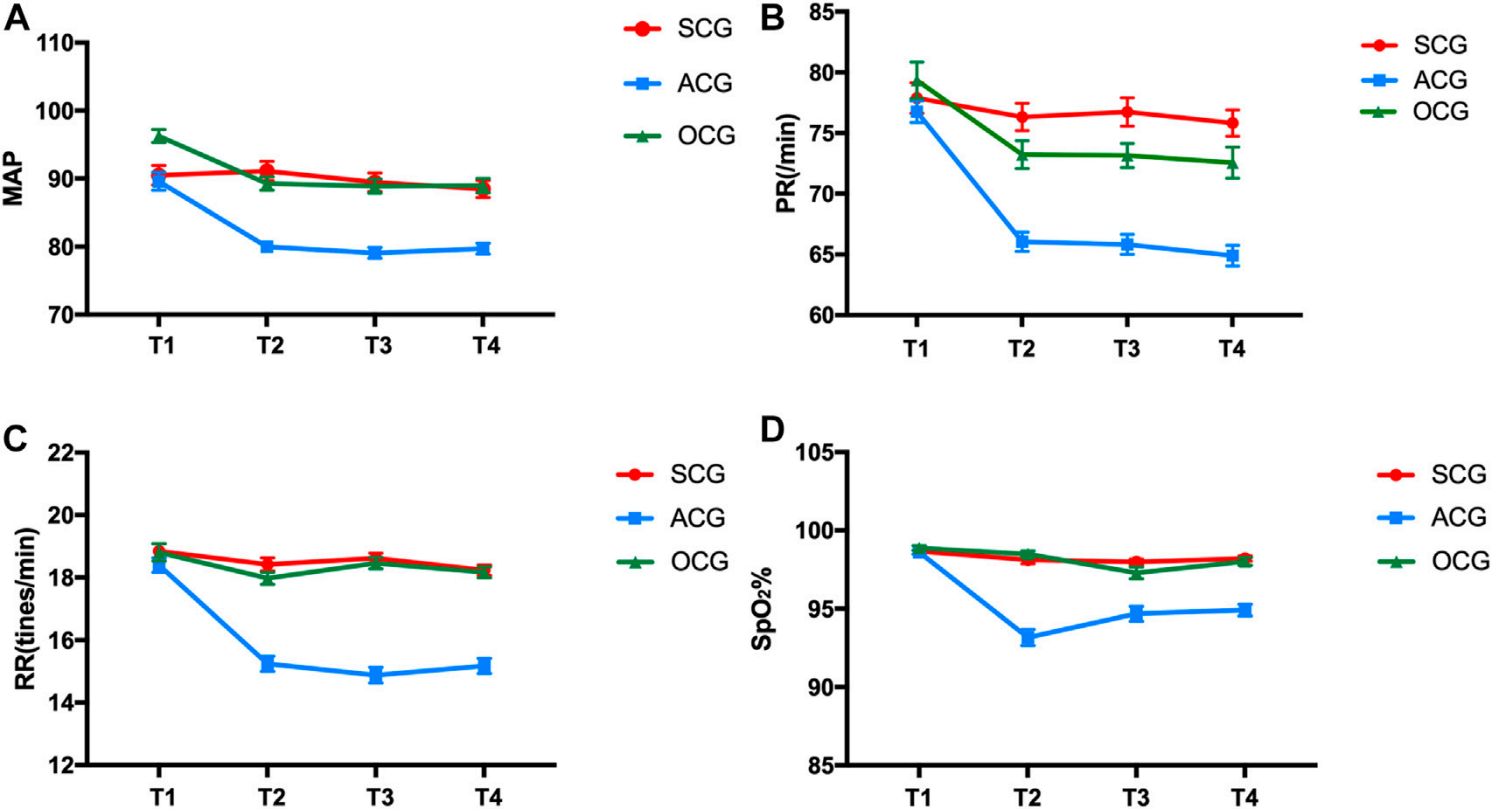
Comparison of MAP **(A)**, PR **(B)**, RR **(C)** and Sp02 **(D)** of patients at different periods during colonoscopy. SCG: sedation and analgesia colonoscopy group, ACG: anesthesia colonoscopy group and OCG: ordinary colonoscopy group.

The patients’ vital signs were recorded at four different time points. Before colonoscopy (T1), the MAP, PR, RR and SpO2 of the three groups were not significantly different (*p* > 0.05). After and during the examination, the MAP, PR, RR and SpO2 at T2, T3 and T4 were within the normal range, but the vital signs of the ACG were significantly lower than those of the SEG and OCG (*p* < 0.05). There was no significant difference in the vital signs of the SCG and OCG at different time points after the examination (as shown in Table 2; Figure 2).

**TABLE 2:** Comparison of vital signs at different time points during colonoscopy (‾χ±s).

	SCG (N = 93)	ACG (N = 90)	OCG (N = 41)	*p*
MAP				
T1	80.51 ± 13.95	89.58 ± 12.49	96.26 ± 13.95	*
T2	91.13 ± 13.39	79.98 ± 7.46	89.28 ± 6.36	#♦
T3	89.48 ± 13.17	79.08 ± 7.46	88.89 ± 6.68	#♦
T4	88.46 ± 12.11	79.73 ± 7.67	88.97 ± 6.92	#♦
PR				
T1	77.88 ± 12.00	76.76 ± 8.38	79.34 ± 9.67	
T2	76.32 ± 10.76	66.06 ± 7.48	73.22 ± 7.30	#♦
T3	76.74 ± 11.18	65.83 ± 7.74	73.15 ± 6.36	#♦
T4	75.82 ± 10.78	64.91 ± 8.06	72.56 ± 8.19	#♦
RR				
T1	18.85 ± 1.56	18.10 ± 2.14	18.81 ± 1.79	
T2	18.41 ± 1.99	15.24 ± 2.30	17.98 ± 1.25	#♦
T3	18.62 ± 1.48	14.88 ± 2.35	18.46 ± 1.19	#♦
T4	18.23 ± 1.59	15.18 ± 2.29	18.17 ± 1.12	#♦
SpO2				
T1	98.68 ± 1.02	98.61 ± 1.13	98.87 ± 0.95	
T2	98.12 ± 2.40	93.18 ± 4.95	98.49 ± 1.31	#♦
T3	97.99 ± 1.79	94.69 ± 4.49	97.29 ± 2.49	#♦
T4	98.02 ± 1.59	94.92 ± 3.56	98.02 ± 1.59	#♦

*Statistically significant difference between the SCG and OCG (*p* < 0.05); # Statistically significant difference between the SCG and ACG (*p* < 0.05); ♦ Statistically significant difference between the OCG and ACG (*p* < 0.05).

**FIGURE 3:**
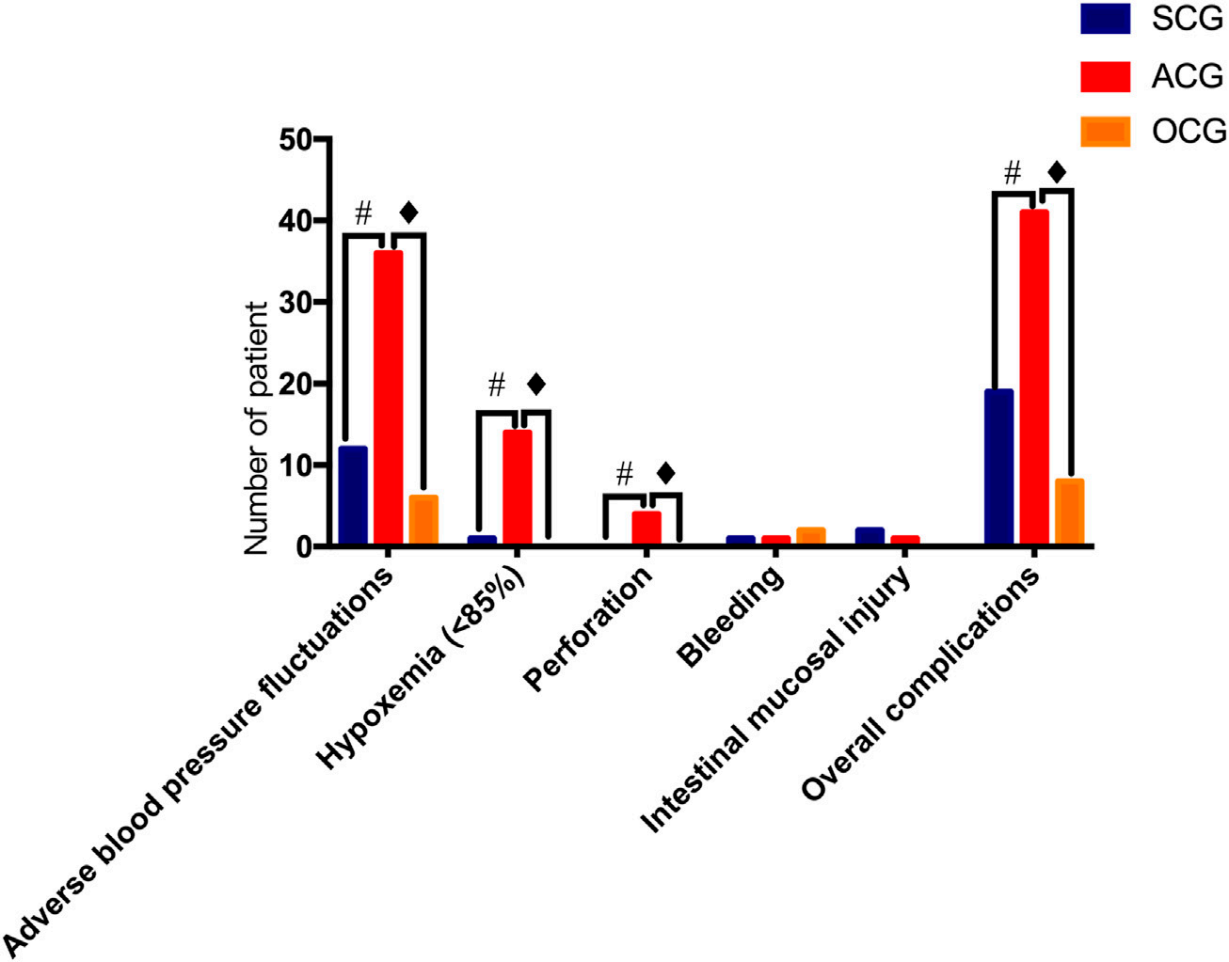
Safety assessment of colonoscopy in the different groups. SCG: sedation and analgesia colonoscopy group; ACG: anesthesia colonoscopy group and OCG: ordinary colonoscopy group. # Statistically significant difference between the SCG and ACG (*p* < 0.05).

The incidence of adverse blood pressure fluctuations in the SCG, ACG and OCG was 17.20% (16/93), 40% (36/90) and 14.63% (6/41), respectively. The incidence of adverse blood pressure fluctuation in the ACG was significantly higher than that in the SCG and OCG (*p* < 0.05), while there was no significant difference between the SCG and OCG (*p* = 0.711). In addition, the number of patients with hypoxemia in the SCG, ACG and OCG was 1, 14 and 0, respectively. The incidence of hypoxemia in the ACG group was significantly higher than that in the other two groups (*p* < 0.05), while the incidence of hypoxemia in the SCG and OCG was not significantly different (*p* = 0.507). There were 4 cases of perforation caused by colonoscopy in the ACG, but there were no cases of perforation caused by colonoscopy in the SCG and OCG, which was statistically significant (*p* < 0.05). There were no significant complications, such as bleeding or mucosal injury, in the SCG, ACG or OCG. The total number of patients who experienced complications in the SCG, ACG, and OCG was 19/93 (20.43%), 41/90 (45.56%) and 8/41 (19.51%), respectively. The total incidence of complications in the ACG was significantly higher than that in the SCG and OCG (*p* < 0.05), while the total incidence of complications in the SCG and OCG was not significantly different (*p* = 0.903). Some patients in the OCG had more than one complication (as shown in Table 3; Figure 3).

**TABLE 3:** Safety assessment of colonoscopy in the different groups (‾χ±s).

	SCG (N = 93)		ACG (N = 90)	OCG (N = 41)	*p*
Adverse blood pressure fluctuations (>20%)		16	36	6	#♦
Hypoxemia (<85%)		1	14	0	#♦
Incidence of perforation		0	4	0	#♦
Incidence of bleeding		1	1	2	
Intestinal mucosal injury		2	1	0	
Overall complications		19	41	8	#♦

*Statistically significant difference between the SEG and OEG (*p* < 0.05); # Statistically significant difference between the SEG and AEG (*p* < 0.05); ♦ Statistically significant difference between the OEG and AEG (*p* < 0.05).

### Patient tolerance

The overall pain score (0.79 ± 1.09) during colonoscopy in the SCG was significantly higher than that in the ACG but significantly lower than that in the OCG (*p* < 0.05). The satisfaction score of the SCG was 9.26 ± 1.16, which was not different from that of the ACG but was significantly higher than that of the OCG (*p* < 0.05). The proportion of patients who would reselect and recommend this examination method in the OCG was 78.05% (32/41), which was significantly lower than that in the SCG [93.55% (87/93)] and ACG [94.44% (85/90)] (*p* < 0.05). The dizziness score of the SCG patients after examination was 0.27 ± 0.55, which was significantly lower than that of ACG patients but significantly higher than that of OCG patients (*p* < 0.05) (as shown in Table 4).

**TABLE 4:** Comparison of patient tolerability among the different groups (‾χ±s).

	SCG (N = 93)	ACG (N = 90)	OCG (N = 46)	*p*
Postresuscitation amnesia				*#♦
Complete memory	69	53	46	
Partial amnesia	24	37	0	
Dizziness score	0.27 ± 0.55	0.67 ± 1.05	0.05 ± 0.22	*#♦
Max pain score during colonoscopy	0.93 ± 1.19	0	3.37 ± 1.28	*#♦
Overall pain score	0.79 ± 1.09	0	2.98 ± 1.27	*#♦
Patient satisfaction	9.26 ± 1.16	9.42 ± 1.41	6.63 ± 1.13	*♦
Choose again	87	85	32	*♦
Recommend to others	87	85	32	*♦

*Statistically significant difference between the SCG and OCG (*p* < 0.05); # Statistically significant difference between the SCG and ACG (*p* < 0.05); ♦ Statistically significant difference between the OCG and ACG (*p* < 0.05).

## Discussion

Colonoscopy is the main method used for diagnosis and treatment of IBD. It can also be used to observe the treatment effect in a long-term follow-up and detect colitis-related tumors (Makkar and Shen, 2013). In addition, therapeutic colonoscopy allows removal of discrete, isolated hyperplastic polyps or adenomatous masses in UC or CD patients and can worsen primary or secondary anastomotic stenosis associated with IBD (Makkar and Shen, 2013). Although the incidence rate of IBD in Asian countries is still lower than that in Western countries, it has increased recently (Doh et al., 2015).

Intestinal perforation is one of the most serious complications of IBD (Brihier et al., 2005). CD is a risk factor related to intestinal perforation caused by colonoscopy (de’Angelis et al., 2018). UC is a dynamic disease that may become active or inactive alternately many times during the course of the patient’s life. In a study involving 1161 patients, half of UC patients were in remission at any given time during the study period (Li et al., 2019). However, even if the disease is inactive for a long time, a long disease duration increases the risk of colon cancer. Therefore, patients with UC need more frequent colonoscopies. In current guidelines, colonoscopy is recommend every 1–2 years for patients with an 8-year or longer history of UC (DiCaprio et al., 2018). IBD patients undergoing colonoscopy should undergo multiple biopsies of the colon to confirm and monitor disease activity (Navaneethan et al., 2011). The risk of perforation is higher due to more frequent colonoscopies and the large number of biopsies of colon lesions. The study found that polypectomy or biopsy during colonoscopy increased the risk of perforation by 1.9 times (Makkar and Shen, 2013).

To date, the research literature on endoscopic perforation in the IBD population is limited and has inconclusive findings. Arora and others used the Medicare database to prove that the perforation rate was 0.06% in IBD outpatients undergoing colonoscopy (Arora et al., 2009), which may be due to the mild condition of the outpatients. In a meta-analysis of an observational study involving 347 CD patients who underwent endoscopic treatment, 14 patients (2%) reported major complications, including 13 patients with an intestinal perforation (Hassan et al., 2007). In a retrospective study of 384 UC patients who underwent colonoscopy, one patient suffered from a perforation (Navaneethan et al., 2011). In a retrospective study involving 33773 IBD patients, 344 patients (1%) had a colon perforation that was caused by colonoscopy (Navaneethan et al., 2011). In addition, another study of 151 colonoscopies and 70 polypectomies performed in patients with IBD did not report any case of bleeding or perforation (Rubin et al., 1999). Three perforations (2 patients with CD and 1 patient with UC) were reported in a study of IBD patients, including 558 patients who underwent colonoscopy (Terheggen et al., 2008). We had 224 patients with IBD, and 4 patients (1.8%) had a colonoscopy-related colon perforation. The reason may be that our clinical center is one of the largest IBD diagnosis and treatment centers in China. Some patients have poor treatment outcomes in other hospitals or are in serious condition, so the perforation rate is relatively higher.

Intestinal perforation may cause intestinal contents to leak into the abdominal cavity or mesenteric space, leading to acute peritonitis. If not found and treated in time, the outcome may be devastating and usually requires emergency surgical intervention. The mortality rate is reported to be 5% (Makkar and Shen, 2013; Doh et al., 2015). With the widespread use of biological agents, immunomodulators and corticosteroids in the treatment of IBD, the consequences of perforation may be more adverse (Navaneethan et al., 2011). Studies have shown that the preoperative use of infliximab in the treatment of UC is associated with an increased risk of developing a postoperative infection (Navaneethan et al., 2011).

The prognosis of surgical treatment for IBD is poor, especially in patients with CD. Such a complex surgery is recommended to be performed by experienced surgeons in an large-volume center (Sampietro et al., 2013). Therefore, a safe and comfortable colonoscopy sedation method is necessary for IBD patients. Our study shows that the moderate sedative and analgesic effect of midazolam and dezocine in patients undergoing colonoscopy is significantly lower than that of intravenous propofol for general anesthesia colonoscopy, and the difference is statistically significant. It may be that the patients under intravenous anesthesia with propofol are unconscious and have no sense of pain, so the pain under colonoscopy cannot be reflected, which is more likely to cause perforation. Sedation and analgesia colonoscopy involves moderate sedation, which allows the patient to respond to severe pain and to communicate, which helps endoscopic doctors better understand the current situation of the patients to reduce the possibility of injury. In addition, during colonoscopy, the doctor using the endoscope found approximately 45%–60% cases of iatrogenic intestinal perforation, especially in patients deeply sedated under intravenous anesthesia. The patient could not give feedback in time, and a considerable number of iatrogenic intestinal perforations were not recognized immediately, leading to further deterioration of the patient’s condition. The related mortality rate could be as high as 5%–25% due to delays in the treatment of intestinal perforation and underlying diseases (de’Angelis et al., 2018). Intravenous propofol for anesthesia improves patient compliance during colonoscopy, but the treatment window following anesthesia induction is narrow, which may lead to complications such as hypoxia, respiratory depression, apnea, hypotension and arrhythmia (das Neves et al., 2016). Even professional doctors with airway management training and clinical experience have difficulty controlling patients (das Neves et al., 2016; Kim et al., 2020). Aguero et al. reported that patients who received large doses of propofol often have hypotension and bradycardia (das Neves et al., 2016). Some experiments have shown that the incidence of adverse blood pressure fluctuations during propofol-induced anesthesia endoscopy is higher (Chen et al., 2022), which is similar to the results of this experiment. Colonoscopy under intravenous anesthesia with propofol leads to lower oxygen saturation in elderly patients (Martínez et al., 2011).

Other studies have reported that patients over 80 years old can reach a level of deep sedation with lower doses of propofol in endoscopic treatment of endoscopic submucosal dissection, but they are more prone to hypoxemia (Gotoda et al., 2016). Some studies have shown that the incidence of complications and hypoxemia following digestive endoscopy under propofol anesthesia in the general population is 38.64% and 13.64%, respectively, which is much higher than that of midazolam combined with dezocine sedation for analgesia digestive endoscopy (Chen et al., 2022).

This experiment suggested that the total complication and hypoxemia rates of propofol anesthesia colonoscopy in IBD patients were 45.5% and 15.6%, respectively, which were significantly higher than those of sedation colonoscopy and ordinary colonoscopy. The reason for the high rates of total complications in this experiment may be that patients with IBD are more prone to bleeding, perforation and other complications during colonoscopy (Navaneethan et al., 2011). The incidence of sedation and analgesia hypoxemia and overall complications is lower than that of propofol-induced anesthesia (Navaneethan et al., 2011). Registered nurses trained in the digestive endoscope system can assist the endoscopy doctor in inducing sedation and analgesia and in the digestive endoscope operation, vital sign monitoring and temporary emergency treatment, which reduces both equipment costs and labor costs (Dossa et al., 2021).

The American Society of Gastrointestinal Endoscopy (ASGE) and the Canadian Association of Gastroenterology (CAG) noted that the combined use of benzodiazepines and opioids is sufficient for inducing sedation and analgesia for gastrointestinal endoscopy, especially for inducing moderate sedation and analgesia during colonoscopy (Byrne et al., 2008; Early et al., 2018; El Shahawy and El-Fayoumy, 2019). According to both GSGMD and SSGE, midazolam is recommended as the first choice benzodiazepine for inducing sedation (Igea et al., 2014; Riphaus et al., 2016). The results of a prospective trial conducted by Christ et al. showed that midazolam reduced the MAP of elderly patients by 10 mmHg on average and did not cause any severe blood pressure fluctuations (Christe et al., 2000). Midazolam has a safe and effective sedative effect, but its analgesic effect is not ideal. Dezocine combined with propofol for anesthesia colonoscopy reduces the occurrence of adverse reactions and increases the safety of the operation (Xu et al., 2016). Compared with fentanyl combined with propofol colonoscopy, dezocine combined with propofol colonoscopy has a lower incidence of adverse reactions during and after the operation and a faster recovery time, as reported by Baykal et al. Our previous studies have shown that midazolam and dezocine are safer for sedation and analgesia colonoscopy in the general population (Chen et al., 2022). Research shows that dezocine can reduce the risk of cardiovascular and respiratory depression, increase the analgesic effect, reduce limb activity, shorten the awakening time, and improve the quality of awakening (Li et al., 2019), which is consistent with this study. Compared with propofol anesthesia colonoscopy, the use of midazolam and dezocine combined with induction sedation and analgesia colonoscopy has fewer complications and better safety. However, there has been no discussion on safe sedation and analgesia for colonoscopy in IBD patients, and more research is needed to guide appropriate and safe sedation and analgesia programs.

Colonoscopy is the main method used for diagnosing and treating IBD. It can also be used to assess the treatment effect in a long-term follow-up and detect colitis-related tumors. If necessary, it can also be used in endoscopic treatment. IBD patients need frequent colonoscopies (Makkar and Shen, 2013), so a comfortable colonoscopy method is very important.

Moderate sedation can relieve discomfort during colonoscopy and improve the patient’s tolerance and acceptance and the success rate of the examination. In contrast, insufficient sedation may cause discomfort and pain in patients, resulting in fear of colonoscopy and poor satisfaction and compliance (El Shahawy and El-Fayoumy, 2019). The comfort requirements during colonoscopy are high, and the quality of sedation is the main indicator of satisfaction. The satisfaction of patients who undergo colonoscopy directly reflects the sedation effect and sedation quality (Kilgert et al., 2014). The European Society of Gastrointestinal Endoscopy (ESGE) (Dumonceau et al., 2015) and SSGE (Igea et al., 2014) both noted that moderate sedation can improve patient satisfaction with colonoscopy. Another study pointed out that moderate sedation can improve not only satisfaction but also patients’ compliance with repeated examinations (Loftus et al., 2013). Previous studies have shown that there is no significant difference between satisfaction and compliance with digestive endoscopy and propofol anesthesia digestive endoscopy in the general population (Chen et al., 2022). A study conducted by Jin et al. observed that for patients receiving sedative and analgesic drugs under digestive endoscopy, even if there are frequent biopsies or longer diagnosis and treatment times, midazolam sedation and active monitoring can improve patient satisfaction (Jin et al., 2017). Our study also showed that there was no significant difference between satisfaction and compliance with colonoscopy under sedation with midazolam and dezocine and digestive endoscopy under analgesia with propofol in patients with IBD, and the satisfaction and compliance rates of both were higher than those of the ordinary endoscopy group.

This study has some limitations. 1) Our research is a nonrandomized, nonblinded study, but we hope that we can carry out randomized trials in the future. 2) This is a single-center study with a small sample size, and the analysis of the related factors and the causes of complications is insufficient. Therefore, large-scale multicenter studies are needed in the future. 3) Most of our questionnaires were completed after the examination, and these answers may have been affected by the use of sedatives, thereby possibly affecting the rate of patient dissatisfaction.

## Conclusion

In conclusion, compared with ordinary endoscopy, analgesia colonoscopy allows the use of midazolam combined with dezocine for sedation and patients are more comfortable, more satisfied and more compliant. In terms of comfort, satisfaction and patient compliance, it is equivalent to propofol anesthesia colonoscopy. In terms of safety, midazolam combined with dezocine sedation and analgesia colonoscopy is equivalent to ordinary endoscopy, and the rates of perforation, hypoxemia and other complications are less than those of propofol anesthesia colonoscopy. Therefore, the application of midazolam combined with dezocine during colonoscopy in patients with IBD is worthy of promotion. However, further experiments are needed.

## Data Availability

The original contributions presented in the study are included in the article/Supplementary Materials, further inquiries can be directed to the corresponding authors.
